# Validation of a survey instrument to assess home environments for physical activity and healthy eating in overweight children

**DOI:** 10.1186/1479-5868-5-3

**Published:** 2008-01-11

**Authors:** Michelle L Gattshall, Jo Ann Shoup, Julie A Marshall, Lori A Crane, Paul A Estabrooks

**Affiliations:** 1 Institute for Health Research, Kaiser Permanente of Colorado, Denver, Colorado, USA; 2 Department of Preventive Medicine and Biometrics, University of Colorado at Denver Health Sciences Center, Denver, Colorado, USA; 3 Department of Human Nutrition, Foods, & Exercise, Virginia Tech, Blacksburg, Virginia, USA

## Abstract

**Background:**

Few measures exist to measure the overall home environment for its ability to support physical activity (PA) and healthy eating in overweight children. The purpose of this study was to develop and test the reliability and validity of such a measure.

**Methods:**

The Home Environment Survey (HES) was developed to reflect availability, accessibility, parental role modelling, and parental policies related to PA resources, fruits and vegetables (F&V), and sugar sweetened drinks and snacks (SS). Parents of overweight children (n = 219) completed the HES and concurrent behavioural assessments. Children completed the Block Kids survey and wore an accelerometer for one week. A subset of parents (n = 156) completed the HES a second time to determine test-retest reliability. Finally, 41 parent dyads living in the same home (n = 41) completed the survey to determine inter-rater reliability. Initial psychometric analyses were completed to trim items from the measure based on lack of variability in responses, moderate or higher item to scale correlation, or contribution to strong internal consistency. Inter-rater and test-retest reliability were completed using intraclass correlation coefficients. Validity was assessed using Pearson correlations between the HES scores and child and parent nutrition and PA.

**Results:**

Eight items were removed and acceptable internal consistency was documented for all scales (α = .66–84) with the exception of the F&V accessibility. The F&V accessibility was reduced to a single item because the other two items did not meet reliability standards. Test-retest reliability was high (r > .75) for all scales. Inter-rater reliability varied across scales (r = .22–.89). PA accessibility, parent role modelling, and parental policies were all related significantly to child (r = .14–.21) and parent (r = .15–.31) PA. Similarly, availability of F&V and SS, parental role modelling, and parental policies were related to child (r = .14–36) and parent (r = .15–26) eating habits.

**Conclusion:**

The HES shows promise as a potentially valid and reliable assessment of the physical and social home environment related to a child's physical activity and eating habits.

## Background

Lifestyle behaviours related to food consumption and physical activity are important causes of unhealthy weight gain in children [1]. However, the current social and physical environment that children encounter at home and school are often counterproductive to promoting healthy eating and physically active lifestyles [2-4]. The effect of the home environment (both physical and social) on these two behavioural areas is a focus of current investigation.

Several investigators have looked at the physical and social environments that might contribute either positively or negatively to physical activity levels in children. Sallis and colleagues examined environmental correlates of physical activity in preschool children and found that convenient play spaces and the frequency and duration of time in play spaces were significantly associated with physical activity [5]. Stucky-Ropp found that for 5^th ^and 6^th ^grade girls, the number of active toys and exercise equipment in the home was related to physical activity [6]. One study by Moore in 1990 using data from the Framingham Children's Study found that children ages 3–7 who have active mothers are 2.0 times as likely to be physically active, 3.5 times as likely to be active if their fathers are active, and 5.8 times as likely to be active if both parents are physically active [7]. Parental support for children's physical activity has also been significant in several studies. Parental support in the form of providing transportation to sports or other physical activities was correlated with increased physical activity in children ages 9 to 14 in several studies [8-10]. In addition, parental verbal encouragement and prompting children to be physically active were also found to be significantly associated with higher physical activity in preschool through adolescent children [5,8,10].

Similar research has also examined the relationship between the social and physical environment on children's eating patterns [11-13]. In a review of family influence on children's eating habits, Baranowski proposed that a child's eating environment includes physical attributes related to availability and accessibility as well as parental behaviours, parental beliefs and knowledge about nutrition and parenting skills [12].

The relationship between food accessibility and consumption is not a new idea. One of the earliest studies of this phenomenon demonstrated that the total number of food items that were in plain view within the house was positively associated with body weight [14]. In a study of fourth and fifth grade children, Kirby and associates demonstrated that regardless of the amount of fruits and vegetables available within the home, unless the food was cleaned, pealed, and within easy reach of the child, children reported that they did not consume them with great regularity [13]. In a study by Hearn and associates, they found that, after controlling for psychosocial characteristics, third grade children reported consuming a greater quantity of fruits and vegetables when fruits and vegetables were routinely situated on the kitchen counter, somewhere in the open, or stored "ready for use" in the refrigerator [11]. More recently, Cullen and Baranowski found that up to 35% of the variability in fourth through sixth grade children's fruit and vegetable consumption was related to the availability and accessibility of fruits and vegetables in their homes [15].

In an attempt to reconcile and test the social and physical home environmental variables on child weight, activity and eating behaviours, Golan and Weizman developed a conceptual model based in social ecological theory, as a framework to guide the treatment of childhood obesity [16]. The model suggests that the parent is the most influential environmental variable related to a child's weight and highlights four areas primarily controlled by parents that influence children's behaviours. The four areas that Golan's model emphasizes are parental knowledge of healthy lifestyle habits, parenting skills, the physical home environment and parental role modelling of a healthy lifestyle [16].

A number of survey measures that assess some aspect of a child's home environment have been developed [17-20]. For example, Golan's Family Eating and Activity Habits Questionnaire measures the social environment of parental authority and family eating style [17]. Karen Cullen developed several food availability questionnaires relating to high-fat, low-fat and fruit/vegetable availability and barriers to healthy eating at home [18]. A Children's Eating Behaviour Questionnaire was developed by Jane Wardle and colleagues to assess a child's habitual eating style for weight related interventions [19]. Similarly, questionnaires to assess a child's physical activity environment have been developed. Clare Hume and associates developed a measure to assess a child's perception of the home and neighbourhood physical activity environment [20]. However, to date, there is a lack of measurement tools to assess both the physical and social environmental components of the home environment that contribute to a child's physical activity and healthy eating that also integrates the components highlighted in previous research.

**Figure 1:**
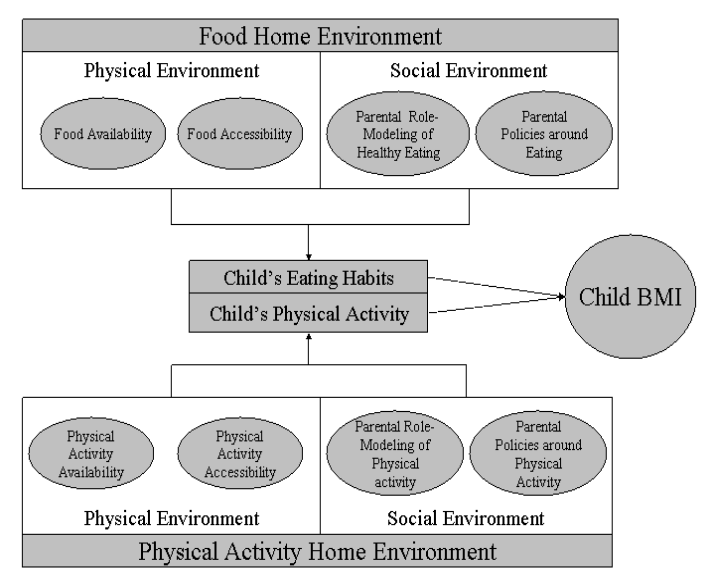
Conceptual Model for Eating and Physical Activity Environmental. Influences in the Home.

The goal of the present study is to develop a survey instrument that can accurately and reliably assess aspects of the home social and physical environment that influence a child's eating and physical activity habits. Figure 1 is a pictorial representation of primary factors to operationalize the home environment based on the extant literature in this area. Specifically, the survey built on and extended Golan's model of environmental influence incorporating the aspects of the home environment that have been significantly correlated to physical activity or eating habits in previous studies.

## Methods

The Kaiser Permanente Colorado Institutional Review Board approved this study and all study participants signed the appropriate forms including parental consent and child assent. The study population consisted of all parents enrolled in *Family Connections*; a two-year randomized controlled trial that evaluated the efficacy of different forms of parental interventions to support child weight management. The data used for the present study includes only the baseline assessments from the Family Connections study. Parents and children were invited to participate in the study if the child's Body Mass Index was at or above the 85^th ^percentile ranking for age and gender, the child was between eight and twelve years old, the parent had the ability to understand spoken and written English, and the parent had primary custody of the child.

Families who agreed to participate in the Family Connections study attended an orientation and baseline assessment visit. Informed consent and child assent were explained and collected at this visit. For the purposes of the Family Connection study, one parent was designated as the primary caregiver based on self-report and ability to commit to attending all intervention sessions and study visits. Each parent and child filled out several self-administered surveys and the child was given an accelerometer to wear for one week to objectively measure their physical activity. The Home Environment Surveys were coded with a non-identifying ID number that allowed for surveys from a given family to be matched during analyses. Participants were provided with a second copy of the Home Environment Survey to take home with instructions to retake the survey seven to ten days after the initial survey and then to return their survey along with the accelerometer in a pre-addressed, pre-paid return envelope provided by study staff.

### Survey Construction and scoring

The Home Environment Survey was comprised of 126 items divided into ten scales. The scales were labeled as: 1) Physical Activity Availability, 2) Physical Activity Accessibility, 3) Fruit/Vegetable Availability, 4) Fruit/Vegetable Accessibility, 5) Fat/Sweet Availability, 6) Fat/Sweet Accessibility, 7) Parental Role Modelling of Physical Activity, 8) Parental Role Modelling of Healthy Eating, 9) Parental Policies to Support Physical Activity, and 10) Parental Policies to support Healthy Eating. Scales ranged from three items (Fruit/Vegetable Accessibility) to 27 items (Fruit/Vegetable Availability).

The Home Environment Survey included items that were developed specifically for the Family Connections study and, when possible, items from previously validated scales [17,21]. Any items from previously validated scales with a self-reported "yes/no" answer were modified by rescaling the question to a five-point scale ("never" to "always") to increase both the potential variability of responses and the sensitivity to change (items taken from validated surveys are listed in Table 1). In the Home Environment Survey, the home social environment was operationalized as parental role modelling and parental policies and skills that are related to healthy eating and physical activity. The home physical environment was operationalized as the availability (physical presence) and accessibility (ease of access) of foods and activity resources.

**Table 1:** Items taken from previously validated surveys

**Items taken from the Food Availability Questionnaire** **Items were modified by changing from Yes/No Answers to a 5 point scale ranging from Never to Always**	**Item Retained in final Home Environment Survey**	**Items taken from the Family and Activity Habits Questionnare** **Items were modified by changing from Yes/No Answers to a 5 point scale ranging from Never to Always**	**Item Retained in Final Home Environment Survey**
**Fruit/Vegetable Availability Scale**		**Fat/Sweets Availability Scale**	
Apples	Yes	Chips	Yes
Applesauce	Yes	Popcorn	Yes
Bananas	Yes	Nuts	Yes
Cantaloupe/Melon	Yes	Crackers	Yes
Fruit Salad	Yes	Sunflower Seeds	Yes
Grapes	Yes	Candy	Yes
Oranges	Yes	Wafers	Yes
Peaches	Yes	Cookies	Yes
Strawberries	Yes	**Healthy Eating Parental Role Modelling Scale**	
Watermelon	Yes	Eat meals in living/TV Area	Yes
Applejuice	Yes	Take second helpings	Yes
Grapejuice	Yes	Eat while standing	Yes
Orangejuice	Yes	Eat from pot/pan	Yes
Broccoli	Yes	Eat while watching TV/reading	Yes
Carrots	Yes	Eat when you were bored	Yes
Cauliflower	Yes	Eat when you were in a bad mood/angry	Yes
Celery	Yes	Eat in a disorderly way	Yes
Corn	Yes	Eat late at night	Yes
Lettuce	Yes	**Healthy Eating Parental Policies Scale**	
Peas	Yes	What do you do when your child is not hungry	No
Green Beans	Yes	Eat breakfast with child	Yes
Potatoes	Yes	Eat lunch with child	Yes
Tomatoes	Yes	Eat pm snack with child	Yes
		Eat dinner with child	Yes
		Can child eat snacks without permission	Yes

Two scoring scales were used for survey responses. For the section on the Availability of Physical Activity resources, a checklist was provided for parents to complete and scores were recorded as either a "0" or "1." A sum score was used for analysis of this section. The remainder of the survey was scored on a scale of 0–4 with a higher score reflecting a more positive response. Items were reverse scored when the question asked about a negative influence on the home environment. For all sections scored on a scale of 0–4, items were summed and divided by the number of items answered for an average summary score for each section. In order to be calculated as a summary score, all sections had to have at least 75% response to items.

### Measures used for validity

Parental physical activity was measured by the Rapid Assessment of Physical Activity Questionnaire (RAPA) [22]. This questionnaire included a nine-item survey that assessed the number of reported days of moderate activity (for at least 30 minutes) and vigorous activity (for at least 20 minutes). For the Family Connections study, the Rapid Assessment of Physical of Physical Activity Questionnaire was modified by adding sub-questions to assess minutes of moderate and vigorous physical activity each day. The Rapid Assessment of Physical Activity Questionnaire has been validated and was comparable with other validated physical activity surveys [23]. The results from the Rapid Assessment of Physical Activity Questionnaire were calculated as total minutes of moderate and vigorous activity per day.

Parent eating habits were assessed by the Fat and Fiber-Related Diet Behaviour Questionnaire (FFB) [24]. Each food item was ranked on a four-point scale of Rarely/Never, Sometimes, Often and Usually. For this survey, a higher score indicated a higher degree of fat in the diet. One continuous summary variable for fat consumption was used in the validity analysis of the Home Environment Survey. In a validation study, the FFB had correlations of .53 for fat intake and .50 for fiber intake with the Food Frequency Questionnaire (which has been validated previously in comparisons with dietary logs and 24 hour dietary recall). Test/retest reliability for the FFB was .74 for the fiber scale and .77 for the fat scale [24,25].

Child physical activity was objectively assessed by accelerometers (model 7164; Actigraph LLC, Pensacola, FL). Children wore the accelerometers from the time they got up in the morning until they went to bed at night for one week. The accelerometer measured a child's vertical acceleration over 30 second epochs of time. In a validation study where the Actigraph accelerometers were compared to energy expenditure as evaluated by respiratory calorimetry, microwave detector, and heart rate telemetry for various activities, the correlation was 0.66. Inter instrument reliability was also assessed and the correlation was 0.88 [26]. For analyzing validity for the availability and accessibility of physical activity on the home environment survey, total minutes of moderate and vigorous activity per day were calculated.

Child eating habits were assessed using the BLOCK Kid's Questionnaire, a food frequency questionnaire for children aged 8 – 17, which is a 77 item questionnaire surveying foods and beverages consumed in the past 7 days [27]. Respondents identified how many days in the past week they ate certain foods ranging from "none" to every day. Respondents were also asked to identify the typical portion size each time they ate a food. Fruit, vegetable, and sugar sweetened drinks and snack consumption were used to determine validity of the Home Environment Survey. In a preliminary validation study of 8–10 year old children, correlations for different categories of foods ranged from .40–.50 (i.e. "fat," "carbohydrate," "fiber") when compared to 24 hour dietary recall [28].

### Statistical Analysis

Items were considered for trimming if they met at least three of the following requirements: low variability in item responses, extreme means on an item (i.e. ceiling effects), low correlation with its own scale and high correlation with other scales. To assess test/retest reliability, parents completed the Home Environment Survey again one to two weeks after the first assessment. Inter-rater reliability was assessed by having both parents of the 47 two-parent participating families fill out the questionnaire. Test-retest and inter-rater reliability were analyzed using intra-class correlations. Internal consistency for all sections except physical activity availability were analyzed using a correlation matrix and by calculating Cronbach's alpha. To analyse validity, each summary score from the Home Environment Survey scales was compared to the appropriate nutrition and physical activity behaviour measures obtained from the RAPA, BLOCK or Accelerometer data from the Family Connections study. Pearson correlations were run for all Home Environment Survey summary score variables. All statistical analysis was done using the Statistical Package for the Social Sciences (SPSS) software.

## Results

### Demographics

Children ranged from 8 to 12 (one child was thirteen due to lag in recruitment and baseline visit) with an average age of 10.5 years old (2). Fifty-four percent were boys. Sixty-three percent of our sample reported their race as white and 24 percent reported their ethnicity as Hispanic. The study population was recruited from children at or above the 85^th ^percentile for Body Mass Index with the great majority of the participants (87%) being above the 95^th ^percentile for Body Mass Index (Table 2).

**Table 2:** Demographics

	**Number (%)**	**Range**	**Mean**
Child Age		8.0 – 13.2	10.6
Child Gender		N.A.	N.A.
Male	118 (52.0%)		
Female	101 (44.5%)		
Child Body Mass Index			
85^th^–95^th ^percentile	28 (12.8%)	19.1 – 47.6	27.1
95^th ^percentile and over	191 (87.2)		
Race and Ethnicity		N.A.	N.A.
White	130(61.3%)		
Black	13 (6.1%)		
Asian	7 (3.3%)		
American Indian	8 (3.8%)		
Latino	50 (23.6%)		
Other	4 (1.2%)		
Primary Parent Age		24–61	39.9
Primary Parent Gender		N.A.	N.A.
Male	21 (9.7%)		
Female	196 (90.3%)		
Primary Parent Body Mass Index			
≤ 24.9	42 (19.2%)	19.3 – 62.1	31.4
25–29.9	73 (33.3%)		
30–34.4	43 (19.6%)		
≥35	61 (27.9%)		
Secondary Parent Age	N.A.	31.0–58.0	42.4
Secondary Parent Gender			
Male	37 (92.5%)		
Female	3 (7.5%)		
Secondary Parent Body Mass Index			
≤ 24.9	7 (16.3%)	21.5 – 55.4	32.4
25–29.9	9 (20.9%)		
30–34.4	12 (27.9%)		
≥35	15 (34.9%)		
Parental Education Level		N.A.	N.A.
Grade school	2 (0.9%)		
High school	77 (35.5%)		
College	105 (48.4%)		
Graduate School	33 (15.2%)		

The primary caregivers in this study were 90% female with an average age of 40 years old. Secondary caregivers were over 90% male and were slightly older with an average age of 42 years old. The average Body Mass Index for both primary and secondary caregivers was over 30 with over 80% of both primary and secondary caregivers being in the overweight or obese categories. Nearly 50% of primary and secondary caregivers had a college education level and nearly 15% of caregivers had a graduate degree indicating that our study sample was likely from a higher socio-economic status (Table 2).

### Item trimming, internal consistency and reliability

A total of 219 surveys were completed by the primary parent for analysis. For inter-rater reliability, 41 dyads of parents who lived in the same home completed the Home Environment Survey for their household. Finally, 156 surveys were returned between one and two weeks for the test-retest reliability study. Tables 3 (physical activity) and 4 (nutrition) provides a summary of each scale within the Home Environment Survey, what items were retained or dropped, inter-rater reliability, test retest reliability, and internal consistency.

**Table 3:** Internal Consistency and Reliability for Physical Activity Scales

**Scale from Home Environment Survey (All items developed for this survey based on literature review)**	**Item Retained in final Home Environment Survey**	**Cronbach's Alpha**	**Inter-rater** Intra-Class Coefficients	**Test-retest** Intra-Class Coefficients
**Physical Activity Availability (Total Scale)**		N.A.	.88	.99
Inside playroom	Yes		.71	.79
Exercise room	Yes		.83	.84
Sandbox	Yes		.91	.92
Driveway	Yes		.79	.86
Play area/yard	Yes		1.0	.43
Exercise equipment in TV area	Yes		.57	.74
Space to play in TV area	Yes		.56	.71
Swing Set	Yes		.88	.95
Bicycle	Yes		1.00	.84
Rollerblades/skates	Yes		.66	.89
Skateboard/scooter	Yes		.72	.86
Jump rope	Yes		.88	.89
Hiking shoes	Yes		.76	.85
Running shoes	Yes		-.08	.76
Basketball hoop	Yes		.89	.93
Baseball equipment	Yes		.50	.78
Racket	Yes		.89	.84
Hockey Equipment	Yes		.95	.96
Balls	Yes		1.00	.43
Pedometer	Yes		.61	.82
Winter Sports Equipment	Yes		.85	.92
Other Physically Active Toys	Yes		.33	.60
**Physical Activity Accessibility (Total Scale)**		.66	.55	.78
How many of your child's active toys are in working condition	Yes		.26	.72
How many of your child's active toys are stored in area child uses them	Yes		.35	.69
How many of your child's active toys does child need help getting out	Yes		.47	.58
How many of your child's active toys are stored out of sight	Yes		.18	.73
**Physical Activity Parental Role Modelling (Total Scale)**		.68	.30	.85
Your child sees you being physically active	Yes		.22	.78
Your child sees you doing house/yard work	Yes		-.29	.61
Your child sees you use Physical activity as relaxation	Yes		.44	.67
Your child sees you on the computer	No		.22	.86
Your child sees you watching TV/movies	No		.42	.75
Your child hears you talk about sports or physical activity	Yes		.16	.73
Your child hears you say you were too tired to be physically active	Yes		.02	.63
How often are you physically active with your child	Yes		.39	.69
**Physical Activity Parental Policies (Total Scale)**		.79	.24	.80
How often do you encourage your child to be physical active	Yes		.08	.62
How often do you transport your child for physical activity	Yes		.48	.79
How often do you send your child outside to play	Yes		.44	.81
How often do you give your child physical activity options	Yes		.28	.61
How often do you praise your child for being physically active	Yes		-.06	.71

**Table 4:** Internal Consistency and Reliability for Nutrition Scales

**Scale from Home Environment Survey (Item source; 1 = Food Availability Questionnaire; 2 = Items developed for this survey from literature review; 3 = Family Eating and Activity Habits Questionnaire)**	**Item Retained in final Home Environment Survey**	**Cronbach's Alpha**	**Inter-rater** Intra-Class Coefficients	**Test-retest** Intra-Class Coefficients
**Fruit/Vegetable Availability – Total Scale**		.84	.60	.82
Apples (1)	Yes		.73	.79
Applesauce (1)	Yes		.75	.90
Bananas (1)	Yes		.65	.85
Cantaloupe/Melon (1)	Yes		.54	.75
Fruit Salad (1)	Yes		.64	.69
Grapes (1)	Yes		.69	.74
Oranges (1)	Yes		.68	.76
Peaches (1)	Yes		.62	.63
Strawberries (1)	Yes		.71	.76
Watermelon (1)	Yes		.66	.74
Other (2)	Yes		.39	.38
Applejuice (1)	Yes		.79	.85
Grapejuice (1)	Yes		.72	.71
Orangejuice (1)	Yes		.75	.83
Fruit juice blend (2)	Yes		.60	.76
Other 100% Fruit juice (2)	Yes		.79	.75
Broccoli (1)	Yes		.77	.85
Carrots (1)	Yes		.59	.78
Cauliflower (1)	Yes		.71	.85
Celery (1)	Yes		.66	.85
Corn (1)	Yes		.67	.78
Lettuce (1)	Yes		.32	.77
Peas (1)	Yes		.78	.87
Green Beans (1)	Yes		.44	.83
Potatoes (1)	Yes		.26	.83
Tomatoes (1)	Yes		.77	.84
Other (2)	Yes		Not calculated due to low n (n = 7)	.55
**Fruit/Vegetable Accessibility – One Item Scale**		N.A.	.50	.49
How often do you store Fruits/Vegetables in a place that is easily seen (2)	Yes		.47	.48
How often do you store Fruits/Vegetables in place that is known but not seen (2)	No		.52	.44
How often do you store Fruits/Vegetables in a hiding place (2)	No		-.10	.01
**Fat/Sweets Availability – Total Scale**		.80	.67	.80
Chips (3)	Yes		.71	.81
Popcorn (3)	Yes		.54	.65
Nuts (3)	Yes		.77	.80
Crackers (3)	Yes		.42	.72
Sunflower Seeds (3)	Yes		.79	.74
Sugared Drinks (2)	Yes		.79	.86
Soda (2)	Yes		.69	.78
Snack bars (2)	Yes		.62	.83
Candy (3)	Yes		.61	.73
Wafers (3)	Yes		.13	.63
Cookies (3)	Yes		.59	.73
Cake (2)	Yes		.63	.66
Chocolate (2)	Yes		.36	.71
Ice cream, frozen desserts (2)	Yes		.67	.80
**Fat/Sweet Accessibility (Total Scale)**		.59	.22	.79
Sugared drinks easily seen (2)	Yes		.46	.73
Sugared drinks not seen (2)	No		.39	.66
Sugared drinks hidden (2)	Yes		-.20	.69
Snack foods easily seen (2)	Yes		.43	.68
Snack foods not seen (2)	No		.51	.50
Snack foods hidden (2)	Yes		.50	.59
**Healthy Eating Parental Role Modelling (Total Scale)**		.73	.54	.82
Eat healthy snacks (2)	Yes		.52	.67
Eat meals in living/TV Area (3)	Yes		.63	.86
Take second helpings (3)	Yes		.37	.64
Eat unhealthy snacks (2)	Yes		.52	.69
Drink sugared drinks (2)	Yes		.45	.83
Eat while standing (3)	Yes		.44	.65
Eat from pot/pan (3)	Yes		.15	.75
Eat while watching (3) TV/reading (3)	Yes		.48	.77
Eat when you were bored (3)	Yes		.19	. 79
Eat when you were in a bad mood/angry (3)	Yes		.50	.78
Eat in a disorderly way (3)	Yes		.33	.67
Eat late at night (3)	Yes		.64	.75
**Healthy Eating Parental Policies (Total Scale)**		.79	.24	.80
What do you do when your child is not hungry (3)	No		.43	.75
Use food as a reward (2)	Yes		.62	.69
Use food as a punishment (2)	No		.34	.69
Prepare meals with child	Yes		.38	.76
Plan meals with child (2)	Yes		.25	.78
Offer healthy snacks (2)	Yes		.68	.69
Eat breakfast with child (3)	Yes		.41	.76
Eat lunch with child (3)	Yes		.09	.73
Eat pm snack with child (3)	Yes		-.41	.65
Eat dinner with child (3)	Yes		.31	.68
Have scheduled meals (2)	Yes		.40	.76
Can child eat snacks without permission (3)	Yes		.67	.63

The Physical Activity Availability scale included 22 items that were tested for inclusion. Because this scale included a checklist of items, Cronbach's alpha was not computed. No items were removed and the test-retest (r = .99) and inter-rater reliability (r = .88) of the scale were both high. The Physical Activity Accessibility Scale included four items. Cronbach's Alpha was .66, which was considered acceptable for a four-item scale. Inter-rater reliability was moderate at .55 indicating some difference between parents' perceptions of the resource accessibility. Test-retest was high (.78). No items were trimmed from this scale.

The Parental Role Modelling of Physical Activity scale included eight items for testing. Two items reflecting role modelling of sedentary behaviour were trimmed because they were not internally consistent. These two items were "How often does your child see you watching TV," and "How often does your child see you on the computer." With these items removed, the internal consistency was adequate (α = .68) and test retest reliability was high (r = .85). As might be expected the inter-rater reliability was modest (r = .30). The Parental Policies to Support Physical Activity scale included five items for testing. The internal consistency for the scale was strong (α = .79) as was the test-retest reliability (r = .80). Again, the inter-rater reliability was modest (r = .24). No items were removed from this scale.

The Fruit/Vegetable Availability scale included 26 items for testing. Three items were considered for possible trimming because of low correlations and/or low variability including "Applesauce," "Other juice" and "Other Vegetables." However, since removing them did not significantly change the internal consistence of the scale and because the "other" categories might be useful in identifying cultural/geographical differences in fruits/vegetable availability these items were retained. The scale's internal consistency (α = .84), inter-rater reliability (r = .60) and test-retest reliability (r = .82) were all acceptable.

For the Fruit/Vegetable Accessibility scale, three items were originally tested for inclusion in the final survey. Of these items, one was trimmed due to low variability (How often do you store Fruits/Vegetables in a hiding place?). A second question was trimmed (How often do you store Fruits/Vegetables in place that was known but not seen?) because it did not correlate well with the other two questions. A single item was retained to reflect this scale due to its face validity (How often do you store fruits and vegetables in a place that is easily seen?). For this item, inter-rater reliability (r = .50) and test-retest (r = .49) were modest.

The Availability of Fat/Sweets included 14 items that were tested for inclusion. The scale had high internal consistency (α = .80), inter-rater reliability (r = .70) and test-retest reliability (r = .80). No items were trimmed from this scale. The Accessibility of Fat/Sweets included six items for testing. Of these items, two were trimmed because they did not correlate well with their scales and in order to increase internal consistency. The two items that were trimmed were "How often do you store soda and sugared drinks in a place that was known but not seen" and "How often do you store high calorie snacks in a place that was known but not seen." Even with the removal of these items the internal consistency of the scale was questionable (α = .59) and the inter-rater reliability was low (r = .22), however test-retest reliability was good (r = .79).

Parental Role Modelling of Healthy Eating, 13 items, had acceptable internal consistency (α = .83), inter-rater reliability (r = .54), and test-retest reliability (r = .82). No items were trimmed from this section. The Parental Policies to Support Healthy Eating scale included 12 items. One item was trimmed for low variability (How often do you use food as a punishment for your child?). Another item was trimmed due to low correlation with the scale (When it is mealtime and your child is not hungry what do you usually do?). One item was tagged for possible trimming due to low variability and a moderate correlation with its scale ("How often do you use foods as a reward for your child"); however, removing it did not significantly raise the Cronbach's Alpha so it was retained. The final scale had high internal consistency (α = .79) and test-retest reliability (r = .80), but low inter-rater reliability (r = .24).

### Validity

Table 5 provides a correlation matrix for the physical activity related variables. Physical Activity Accessibility (r = .15), Parental Role Modelling (r = .14) and Parental Policies (r = .21) all show small but significant correlations with the child's physical activity as assessed via the accelerometer. Similarly, Physical Activity Parental Policies (r = .16), Parental Role Modelling (r = .31) and Accessibility (r = .15) all had significant correlations with parent self-reports of physical activity.

**Table 5:** Validity Correlation Matrix for Physical Activity

	**Parent Physical Activity (RAPA)**	**Physical Activity Availability**	**Physical Activity Accessibility**	**Physical Activity Parental Role Modelling**	**Physical Activity Parental Policies**
Child Physical Activity (Accelerometer)	.10	.07	.15*	.14*	.21**
Parental Physical Activity (RAPA)		.09	.15*	.31**	.16*
Physical Activity Availability			.33**	.25**	.18**
Physical Activity Accessibility				.28**	.31**
Physical Activity Parental Role Modelling					.49**

* Significant at 0.05

** Significant at 0.01

Table 6 includes the data used to assess the Home Environment Survey validity related to nutrition outcomes. For child fruit consumption, Fruit and Vegetable Availability (r = .23), Accessibility (r = .17), Parental Role Modelling (r = .21), and Parental Policies (r = .28) were all significant correlates. For child vegetable consumption, Fruit and Vegetable Availability (r = .22), Parental Role Modelling (r = .14) and Parental Policies (r = .36) were significant correlates. For parents, the amount of fat in the diet was significantly correlated with fruit and vegetable Availability (r = .15), Role Modelling of Healthy Eating (r = -.26) and Parental Policies to support Healthy Eating (r = -.17). Negative correlations suggest that as parent fat consumption decreases, parental role modelling and healthy food policy scores increase.

**Table 6:** Validity Correlations for Fruit/Vegetable Consumption

	Child Frequency of Fruits (BLOCK)	Parent Amount of Fat in Diet (FFB)	Fruit/Vegetable Availability	Fruit/Vegetable Accessibility	Healthy Eating Parental Role Modelling	HealthyEating Parental Policies
Child Serving of Vegetables (BLOCK)	.32**	-.07	.22**	.05	.14*	.36**
Child Frequency of Fruits (Block)		-.02	.23**	.17*	.21**	.28**
Parent Amount of Fat in Diet (FFB)			.15*	-.05	-.26**	-.17*
Fruit/Vegetable Availability				.19**	.34**	.46**
Fruit/Vegetable Accessibility					.15*	.29**
Healthy Eating Parental Role Modelling						.52**

*Significant at 0.05

** Significant at 0.01

For the child's percent of kilo-calories from sweets, Fat/Sweet Accessibility (r = -.14), Parental Role Modelling (r = -.17) and Parental Policies (r = .-.17) all had small but significant correlations (Table 7). For the parent amount of fat in diet, Fat/Sweet Accessibility (r = .21), Parental Role Modelling (r = -.26) and Parental Policies (r = -.23) all had significant correlations. For both child percent of kilocalories from sweets and parent amount of fat in diet, the correlations with Fat/Sweet Accessibility were opposite of what was hypothesized.

**Table 7:** Validity Correlations for Fat/Sweet Consumption

	Parent Amount of Fat in Diet (FFB)	Fat/Sweet Availability	Fat/Sweet Accessibility	Healthy Eating Parental Role Modelling	Healthy Eating Parental Policies
Child % of Kilo-calories from Sweets (BLOCK)	.14*	-.06	-.14*	-.17*	-.17*
Parent Amount of Fat in Diet (FFB)		-.13	-.20**	-.26**	-.17*
Fat/Sweet Availability			.60**	.28**	.23**
Fat/Sweet Accessibility				.24**	.29**
Healthy Eating Parental Role Modelling					.52**

*Significant at 0.05

**Significant at 0.01

## Discussion

Overall, the Home Environment Survey showed consistency with previous research on childhood nutrition and physical activity and their relationship to the home environment. Parental role modelling of physical activity, parental policies to support physical activity in children and the availability of physical activity toys all showed correlations with child physical activity which supports previous research. Child nutrition findings were also consistent with the previous research that links child nutrition to family eating policies, parental role modelling and the availability and accessibility of foods in the home. In addition, the concepts of availability, accessibility, parental role modelling and parental policies correlate with each other.

In comparison with other surveys measuring child eating patterns, home environment or physical activity environment, the Home Environment Survey showed comparable internal consistency and reliability. The physical activity home and neighborhood environment questionnaire developed by Hume had similar test-retest correlations and internal consistency (e.g., α = .43–.77) for scaled responses. In Golan and Weisman's Family Eating and Activity Habits Questionnaire, internal consistency and test-retest reliability were high. The diet related psychosocial questionnaire developed by Hume showed similar Cronbach's Alpha scores for food availability scales and also showed slightly smaller correlations between the food availability scales and reported food intake.

Although most scales on the Home Environment Survey demonstrated good internal consistency and reliability, scores were lower than expected for Fruit/Vegetable Accessibility and Fat/Sweet Accessibility. These questions were very broad (i.e. "How often do you store high-calorie foods in a place that was known but not seen?") and developed so that there would be consistency across behaviours. From participant feedback while taking the survey, many thought it would be better to make these questions very specific such as "How often are fruits kept in a fruit bowl on the kitchen counter or table?"

For the inter-rater reliability, the vast majority of "primary" caregivers were mothers who also said that they were the primary person who prepared food, planned activities and had the most knowledge of their child's eating and physical activity. With this in mind, inter-rater reliability was still quite high on most sections of the Home Environment Survey. Fat/Sweet Accessibility had a low inter-rater reliability most likely because of the broad questions that were open to interpretation. Physical Activity Role Modelling and Physical Activity Parental Policies both had much lower inter-rater reliability; however, this should be expected because physical activity is often an individual choice and it is likely that two parents could be very different in their amount of physical activity and in their parental policies to encourage their children to be physically active. Healthy Eating Role Modelling and Healthy Eating Parental Policies did have reasonably high inter-rater reliability indicating that nutrition is likely a "family affair." Given that the same foods are available to the entire family and that families often eat together it is reasonable to think that parents would have more similar nutrition role modelling and parental policies around food. Although not within the scope or data available for this study, an interesting future area of research could consider the impact of having more homogeneous or heterogeneous parent perceptions of role modelling and parental policies on child behaviour.

Most scales in the Home Environment Survey did show significant correlations with the appropriate child physical activity/nutrition or parental physical activity/healthy eating variables. However, the availability of physical activity equipment/spaces did not significantly correlate with a child's physical activity. This may be a case where availability is necessary but not sufficient by itself without accessible equipment or play spaces to encourage physical activity. Similarly, accessibility of vegetables was not significantly correlated to child vegetable consumption.

For child percentage of kilocalories from sweets, the fact that availability of fats/sweets was not significant and that accessibility was correlated significantly but in the opposite direction of what was hypothesized, may indicate that other environments play a larger role in fat/sweet consumption for children. Many parents commented that children were often getting sweet/high fat foods at school or activities away from the home. In a study of European children, children reported that fruits/vegetables were not as available when they were outside of the home and that high calorie snacks were more available outside the home lending support to this hypothesis [29].

Parental physical activity was correlated to physical activity role modelling and parental policies; however, the correlation between role modelling and parental physical activity was only moderate. This is likely because the role modelling items on the Home Environment Survey referred to physical activity that was directly observed by the child. Many parents who were regular exercisers reported that they exercised early in the morning or at work when their children could not directly observe their physical activity.

Three main limitations apply to this study. First, all children participating in this study had a Body Mass Index at or above the 85^th ^percentile placing them at risk of overweight or overweight status. This validation study was not able to compare home environments of families whose children were at a healthy Body Mass Index. If children with healthy Body Mass Indices had been included, the ability to detect differences in the home environment between the two groups would have added to the validity testing of the Home Environment Survey survey. The second limitation of this study was that it cannot be generalized to all populations since all study participants were drawn from the Kaiser Permanente membership in one geographic region of the United States. Third, this study is a cross-sectional study using baseline data and so cannot assess the ability of the survey instrument to detect change over time.

Future investigations are necessary to test the survey in a larger sample to allow for the use of confirmatory factor or latent modelling to determine the strength of the factor structure proposed by the Home Environment Survey. Many parents reported that they felt that older children (11 and 12) were less influenced by the home environment as they spent more time at school and out with friends. Testing this survey in several different age populations would be helpful to determine if this survey is equally valid in all age groups. Finally, we had originally intended to include sedentary behaviour as reverse scored items within the physical activity sections; however, it became clear in attempting to score these items and in preliminary analysis that sedentary behaviour and physical activity are related but separate domains. The questions we had included within the physical activity availability, accessibility and parental role modelling sections did not correlate well with the other questions in their scales. In retrospect, we believe it would have been better to have separate scales for sedentary activity. We had also originally included a parental policies section that asked about parental limits for sedentary behaviours. We had an insufficient response rate for the section on parental limits for sedentary behaviours perhaps because parents had a difficult time quantifying the amount of time children are allowed to spend on sedentary activities.

## Conclusion

In conclusion, the Home Environment Survey was designed to give a comprehensive overview of a child's home environment based on a socio-ecological framework. In this pilot study, the Home Environment Survey shows promise as a useful tool for assessing an overweight child's home environment.

## Competing interests

The author(s) declare that they have no competing interests.

## Authors' contributions

MG conducted the literature review, drafted the questions for the Home Environment Survey, collected data, conducted the analysis and drafted the manuscript; PE served as the mentor for this study, directed the conception and inclusion of items in this survey, gave guidance in analysis and critically reviewed the manuscript; JAS was the project manager for the Family Connections study and helped with survey design, data collection, created the final dataset and critically reviewed the manuscript; JM and LC participated in developing the content and scoring of the survey, critically reviewed the analytic procedures used and critically reviewed the manuscript. All authors have approved the submission of this manuscript.

## References

[B1] American Academy of Pediatrics (2003). Policy statement: prevention of pediatric overweight and obesity. Pediatrics.

[B2] Richter KP, Harris KJ, Paine-Andrews A, Fawcett SB, Schmid TL, Lankenau BH, Johnston J (2000). Measuring the health environment for physical activity and nutrition among youth: A review of the literature and applications for community initiatives. Preventive Medicine.

[B3] French SA, Story M, Jeffery RW (2001). Environmental influences on eating and physical activity. Annual Review of Public Health.

[B4] Dowda M, Ainsworth BE, Addy CL, Saunders R, Riner W (2001). Environmental influences, physical activity, and weight status in 8- to 16-year-olds. Arch Pediatr Adolesc Med.

[B5] Sallis JF, Nader PR, Broyles SL, Berry CC, Elder JP, McKenzie TL, Nelson JA (1993). Correlates of physical activity at home in Mexican-American and Anglo-American preschool children. Health Psychology.

[B6] Stucky-Ropp RC, DiLorenzo TM (1993). Determinants of exercise in children. Preventive Medicine.

[B7] Moore JL, Lombardi DA, White MJ, Campbell JL, Oliveria SA, Ellison RC (1991). Influence of parents' physical activity levels on activity levels of young children. The Journal of Pediatrics.

[B8] Sallis JF, Alcaraz JE, McKenzie TL, Hovell MF (1999). Predictors of change in children's physical activity over 20 months. American Journal of Preventive Medicine.

[B9] Sallis JF, Alcaraz JE, McKenzie TL, Hovell MF, Kolody B, Nader PR (1992). Parental behavior in relation to physical activity and fitness in 9-year-old children. Am J Dis Child.

[B10] Hoefer WR, McKenzie TL, Sallis JF, Marshall SJ, Conway TL (2001). Parental provision of transportation for adolescent physical activity. American Journal of Preventive Medicine.

[B11] Hearn MD, Baranowski T, Baranowski J, Doyle C, Smith M, Lin LS, Resnicow K (1998). Environmental influences on dietary behaviour among children: availability and accessibility of fruits and vegetables enable consumption. Journal of Health Education.

[B12] Baronowski T, Smith M, Hearn MD, Lin LS, Baranowski J, Doyle C, Resnicow K, Wang DT (1997). Patterns in children's fruit and vegetable consumption by meal and day of the week. Journal of the American College of Nutrition.

[B13] Kirby SD (1995). Children's fruit and vegetable intake: Socioeconomic, adult-child, regional, and urban-rural influences. Journal of Nutrition Education.

[B14] Coates TJ, Jeffery RW, Wing RR (1978). The Relationship between persons' relative body weights and the quality and quantity of food stored in their homes. Addictive Behaviours.

[B15] Cullen KW, Baranowski T, Owens E, Marsh T, Rittenberry L, De Moor C (2003). Availability, accessibility, and preferences for fruit, 100% fruit juice, and vegetables influence children's dietary behaviour. Health Education and Behaviour.

[B16] Golan M, Weizman A (2001). Familial approach to the treatment of childhood obesity: conceptual model. J Nutr Educ.

[B17] Golan M, Weizman A, Apter A, Fainaru M (1998). Parents as the exclusive agents of change in the treatment of childhood obesity. American Journal of Clinical Nutrition.

[B18] Cullen KW, Klesges LM, Sherwood NE, Baranowski T, Beech B, Pratt C, Zhou A, Rochon J (2004). Measurement characteristics of diet-related psychosocial questionnaires among African-American parents and their 8- to 10-year-old daughters: results from the Girls' health Enrichment Multi-site Studies. Preventive Medicine.

[B19] Wardle J, Guthrie CA, Sanderson S, Rapoport L (2001). Development of the Children's Eating Behaviour Questionnaire. J Child Psychol Psychiat.

[B20] Hume C, Ball K, Salmon J (2006). Development and reliability of a self-report questionnaire to examine children's perceptions of the physical activity environment at home and in the neighbourhood. Int J Behav Nutr Phys Act.

[B21] Marsh T, Cullen KW, Baranowski T (2003). Validation of a fruit, juice, and vegetable availability questionnaire. J Nut Ed Behaviour.

[B22] http://depts.washington.edu/hprc/publications/rapa.htm.

[B23] Topolski TD, LoGerfo J, Patrick DL, Williams B, Walwick J, Patrick MB (2006). The Rapid Assessment of Physical Activity (RAPA) among older adults. Prev Chronic Dis.

[B24] Kristal AR, Shattuck AL, Henry HJ (1990). Patterns of dietary behavior associated with selecting diets low in fat: reliability and validity of a behavioral approach to dietary assessment. J Am Diet Assoc.

[B25] Shannon J, Kristal AR, Curry SJ, Beresfird SA (1997). Application of a behavioral approach to measuring dietary change: the fat- and fiber-related diet behavior questionnaire. Cancer Epidemiol Biomarkers Prev.

[B26] Puyau MR, Adolph AL, Vohra FA, Butte NF (2002). Validation and calibration of physical activity monitors in children. Obesity Research.

[B27] http://www.nutritionquest.com/products/questionnaires_screeners.htm.

[B28] Block G, Murphy M, Roullet P, Wakimoto P, Crawford PB, Block T (2001). Pilot validation of a FFQ for children 8–10 years. Manuscript for Fourth International Conference on Dietary Assessment Methods.

[B29] Sandvik C, De Bourdeaudhuij I, Due P, Brug J, Wind M, Bere E, Perez-Rodrigo C, Wolf A, Elmadfa I, Thorsdottir I, Vas de Almeida MD, Yngve A, Klepp KI (2005). Personal and environmental factors regarding fruit and vegetable intake among schoolchildren in nine European countries. Annals of Nutrition & Metabolism.

